# Testicular Rupture Following Blunt Scrotal Trauma

**DOI:** 10.1155/2019/7058728

**Published:** 2019-12-18

**Authors:** Derek Blok, Matthew Flannigan, Jeffrey Jones

**Affiliations:** Spectrum Health—Michigan State University Emergency Medicine Residency Program, Grand Rapids, MI, USA

## Abstract

Testicular rupture after blunt scrotal trauma is characterized by rupture of the tunica albuginea and extrusion of seminiferous tubules. This is a serious injury and appropriate evaluation and management are necessary both for symptom control, but also for preservation of the testicle. Clinical examination of the scrotum following trauma is difficult and may result in incorrect triage of patients for surgical exploration. This case study describes the assessment and management of blunt testicular trauma in an adolescent lacrosse player.

## 1. Introduction

Blunt scrotal trauma is a common occurrence in male athletes, but serious injuries are rare despite the vulnerable position of the testicles. The cremasteric reflex, testicular mobility, and the strength of the tunica albuginea all contribute to making testicular trauma uncommon [1]. With the increased use of protective athletic cups, the rates of serious injury have also been reduced. Testicular rupture is a rare but serious injury that is characterized by a rip or tear in the tunica albuginea resulting in extravasation of seminiferous tubules. The right testis is injured more often than the left one, because of its greater propensity to be trapped against the pubis or inner thigh [2]. Studies revealed that a 50 kg direct force is required to cause testicular rupture [1]. Severity may vary from a small laceration with minimal extravasation to complete parenchymal destruction [2]. Testicular rupture is a rare but serious injury and appropriate evaluation and timely management are necessary both for symptom control, but also for preservation of the testicle [3]. However, clinical signs may be inconsistent, and investigations are not always definitive in establishing the diagnosis [4].

## 2. Case Presentation

A 15-year-old healthy male was hit in the scrotum by a lacrosse ball. He had immediate, severe pain associated with vomiting. The pain symptoms slowly resolved but he presented to the emergency department (ED) 4 days later with persistent swelling and purple discoloration of the left scrotum. He denied dysuria, hematuria, voiding difficulties, or abdominal symptoms. He did not sustain any other injuries. On examination, this was a well-developed male in no acute distress. Genitourinary examination revealed Tanner stage 4 male genitalia with circumcised phallus. There was no penile swelling or urethral discharge. The left hemiscrotum was approximately 4 times the size of the right, with ecchymosis along the inferior aspect of the scrotum. The left testicle was difficult to palpate due to the extensive swelling and tenderness. The right testicle was normal in size and lay with no palpable abnormalities. Cremasteric reflex was not elicited on the left side.

**Figure 1:**
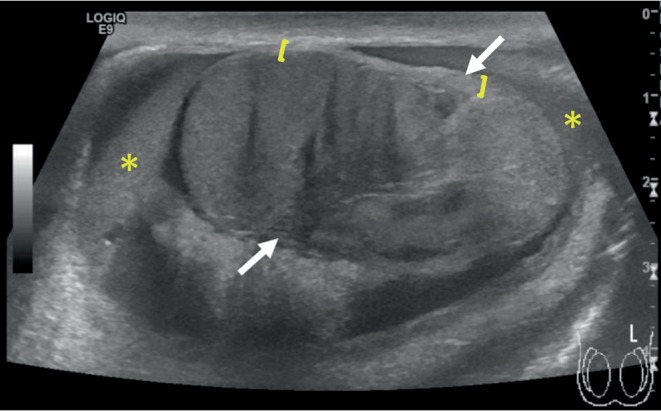
Left testicle sagittal plane. There is abnormal tunica albuginea thickening ([) without obvious disruption, a moderate-sized hematocele (∗), and a linear hypoechoic fracture line (between white arrows) separating a heterogenous appearing testicular parenchyma. Normal testicular parenchyma is present to the left with an abnormal appearance testicle to the right of the fracture line, consistent with an intratesticular contusion.

**Figure 2:**
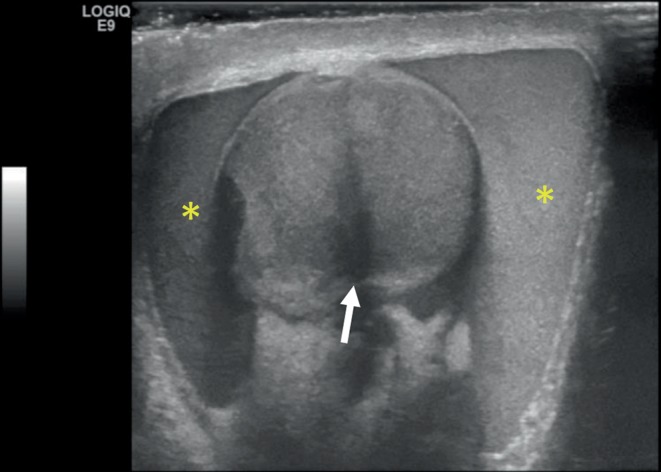
Left testicle mid-transverse plane. Complex fluid collection with low level echoes surrounding the left testicle consistent with a hematocele (∗) with hypoechoic testicular echogenicity consistent with a fracture line (arrow).

Scrotal ultrasonography was performed by the emergency physician at the bedside. This showed moderate scrotal edema on the left with testicular contusion and hematocele. Thickening of the tunica albuginea and subtle contour deformity were consistent with left testicular fracture (Figures 1 and 2). There was no evidence of torsion or infarction.

**Figure 3:**
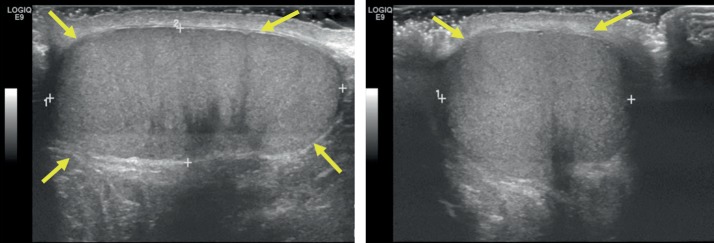
Right testicle in sagittal and transverse planes. Thin tunica albuginea uniformly surrounding testicle (yellow arrows), homogenous testicular parenchyma echogenicity, and absent hematocele.

Urology consultation was requested, and a formal ultrasound ordered, which confirmed the diagnosis of testicular rupture. Color Doppler images on the formal ultrasound showed normal flow to both testicles. The patient was admitted for emergency exploration of his left hemiscrotum due to the ultrasound findings. Intraoperatively, a large hematocele was evacuated, and closer inspection of the testicle revealed a tunica albuginea tear at the upper pole with a significant number of extruded necrotic seminiferous tubules. All of the devascularized extruded tubules were excised. Primary closure of the tunica albuginea was not possible because of edema, so a tunica vaginalis flap was then used to repair the tunica albuginea defect. Postoperatively, there were no complications. Six months later, the patient had a normal scrotal exam on follow-up, and he was advised on the importance of wearing a protective athletic cup (Figure 3).

## 3. Discussion

Previously reported rates of testicular injury with sports participation may underestimate the prevalence of these injuries among adolescent and young adult athletes among whom testicular protective equipment is infrequently used. Any kind of contact sport, without the use of protective aids, may be associated with genital trauma [4, 5].

Examples include a kick to the groin or a bicycle injury. In a survey of 731 male high school and college athletes, 18% experienced a testicular injury during sports, and 36% observed injuries in other team members [5]. Only 12.9% of respondents reported use of athletic cups. The prevalence of testicular injuries was highest in lacrosse (49%), followed by wrestling (33%), baseball (21%), and football (18%).

The differential diagnosis of scrotal injuries is myriad and includes epididymitis, orchitis, incarcerated inguinal hernia, testicular infarction, testicular fracture or rupture, testicular torsion, appendicular torsion, dislocated testes, hydrocele, or hematocele [6]. Clinical examination may be difficult—patients are often reluctant to be examined due to pain and soft tissue swelling can make the testes difficult to palpate [3]. Signs and symptoms requiring further evaluation include significant swelling, ecchymosis, ongoing pain, abnormal “lie” of the testicle, and loss of testicular contours on physical exam. However, testicular rupture may be present with minimal or no pain, and the clinical findings are frequently unreliable in predicting the severity of injury [3, 7]. Despite these limitations, accurate triage of patients requiring urgent surgery is necessary to maximize the opportunity for testicular salvage in severe injuries.

Current American Urologic Association guidelines support ultrasound (US) in blunt scrotal trauma [8]. The main goal of US is to assess the vascular perfusion and integrity of the testes and distinguish testicular rupture from other injuries. Using modern ultrasound equipment, a sensitivity, and specificity of 95–100% for diagnosing testicular rupture is now possible [6, 9]. The normal tunica albuginea in US appears as a thin echogenic line surrounding the testicular parenchyma and is challenging to appreciate especially in the presence of scrotal contusion [10]. Acute bleeding or contusion of the testicular parenchyma typically appears as a hyperechoic area, whereas old blood may be hypoechoic. Heterogeneous parenchyma echotexture and irregular margins are typical characteristics of testicular rupture. Additional sonographic findings include disruption of the tunica vaginalis, fracture lines through the testicle, decreased or loss of blood flow on color Doppler sonography, scrotal thickening, and hematocele formation [3, 6, 10]. Doppler imaging plays an important role in directing management; the presence of vascularity within the testicular parenchyma is indicative of its salvageability [3]. Some false-negative testicular rupture cases diagnosed by US have been reported, leading to misdiagnosis, and delayed surgical intervention [6]. Other imaging studies, such as contrast-enhanced US, nuclear imaging, or magnetic resonance imaging (MRI), have been used to obtain additional information in equivocal cases [10]. Ultimately, testicular rupture is a surgical diagnosis. Persistent symptoms or a concerning examination along with a corresponding history, regardless of imaging findings, requires operative exploration [1].

Point-of-care ultrasound (POCUS) has increasingly been used for early investigation of the acute scrotum [4]. However, the literature is limited to case reports, and two retrospective studies [1, 4, 5, 9, 11]. Using a high frequency linear array probe, the testes should be individually visualized. Identification of the presence of a heterogeneous echo pattern of the testicular parenchyma, with a loss of definition of the outer contour, is highly correlative with testicular injury [12].

Current management strategy for testicular rupture is surgical exploration and repair within 72 hours. Surgery includes evacuation of the hematocele, debridement, and primary closure of the tunica albuginea. Surgical delay may decrease the salvage rate from 80–90% to 45–55% [1]. Additional benefits from early surgical intervention may include preserving testicular function, quicker symptom control, shorter hospital stay, and earlier return to play. However, there is no evidence to suggest inferior outcomes following the conservative management of testicular trauma. Cubillos et al. conducted a study to confirm efficacy of a conservative approach to testicular rupture after blunt scrotal trauma in 7 adolescent boys [12]. Their results showed that neither testicular atrophy nor abnormal echogenicity were detected by US in 6-months follow-up, and only 1 patient needed surgery to relieve a hydrocele 4 months after trauma. Conservative regimen includes scrotal support, proper pain control, ice pack, bed rest, and serial ultrasound [1].

In summary, scrotal ultrasound is the first line imaging modality for the diagnosis of testicular trauma. The ultrasound findings of testis rupture include focal parenchymal echo texture heterogeneity due to hematoma or contusion, discrete intraparenchymal fracture plane, outer testicular contour abnormality, disruption of the tunica albuginea, and hematocele formation. In the setting of conservative management follow-up, ultrasound is also vital to evaluate for an enlarging hematoma. Current management strategy for testicular rupture is surgical exploration and repair, ideally within 72 hours to maximize salvage.
